# A Family of Diverse Kunitz Inhibitors from *Echinococcus granulosus* Potentially Involved in Host-Parasite Cross-Talk

**DOI:** 10.1371/journal.pone.0007009

**Published:** 2009-09-17

**Authors:** Silvia González, Martín Fló, Mariana Margenat, Rosario Durán, Gualberto González-Sapienza, Martín Graña, John Parkinson, Rick M. Maizels, Gustavo Salinas, Beatriz Alvarez, Cecilia Fernández

**Affiliations:** 1 Cátedra de Inmunología, Facultad de Química, Universidad de la República, Montevideo, Uruguay; 2 Laboratorio de Enzimología, Facultad de Ciencias, Universidad de la República, Montevideo, Uruguay; 3 Unidad de Bioquímica y Proteómica Analíticas, Institut Pasteur de Montevideo and Instituto de Investigaciones Biológicas Clemente Estable, Uruguay; 4 Unidad de Bioinformática, Institut Pasteur de Montevideo, Montevideo, Uruguay; 5 Program in Molecular Structure and Function, Hospital for Sick Children, Toronto, Canada; 6 Institute of Immunology and Infection Research, University of Edinburgh, Edinburgh, United Kingdom; New England Biolabs, United States of America

## Abstract

The cestode *Echinococcus granulosus*, the agent of hydatidosis/echinococcosis, is remarkably well adapted to its definitive host. However, the molecular mechanisms underlying the successful establishment of larval worms (protoscoleces) in the dog duodenum are unknown. With the aim of identifying molecules participating in the *E. granulosus*-dog cross-talk, we surveyed the transcriptomes of protoscoleces and protoscoleces treated with pepsin at pH 2. This analysis identified a multigene family of secreted monodomain Kunitz proteins associated mostly with pepsin/H^+^-treated worms, suggesting that they play a role at the onset of infection. We present the relevant molecular features of eight members of the *E. granulosus* Kunitz family (*Eg*KU-1 – *Eg*KU-8). Although diverse, the family includes three pairs of close paralogs (*Eg*KU-1/*Eg*KU-4; *Eg*KU-3/*Eg*KU-8; *Eg*KU-6/*Eg*KU-7), which would be the products of recent gene duplications. In addition, we describe the purification of *Eg*KU-1 and *Eg*KU-8 from larval worms, and provide data indicating that some members of the family (notably, *Eg*KU-3 and *Eg*KU-8) are secreted by protoscoleces. Detailed kinetic studies with native *Eg*KU-1 and *Eg*KU-8 highlighted their functional diversity. Like most monodomain Kunitz proteins, *Eg*KU-8 behaved as a slow, tight-binding inhibitor of serine proteases, with global inhibition constants (*K*_I_^*^) *versus* trypsins in the picomolar range. In sharp contrast, *Eg*KU-1 did not inhibit any of the assayed peptidases. Interestingly, molecular modeling revealed structural elements associated with activity in Kunitz cation-channel blockers. We propose that this family of inhibitors has the potential to act at the *E. granulosus*-dog interface and interfere with host physiological processes at the initial stages of infection.

## Introduction

*Echinococcus granulosus* is a member of a major, though neglected class of helminth parasites, the cestodes. It is the agent of a medically and economically important cosmopolitan zoonosis, with endemic foci in every inhabited continent [1]. This organism requires two mammals for completion of its life cycle: in intermediate hosts (various ungulates, mostly domestic cattle and also humans), eggs develop into metacestodes (hydatid cysts) containing larval worms (protoscoleces) at visceral sites. When the canine definitive host ingests infected flesh, protoscoleces evaginate and attach to the mucosa of the dog duodenum, where they develop to hermaphroditic adult worms producing eggs over a period of several weeks. In dogs, the infection is referred to as echinococcosis.

*E. granulosus* is extremely well adapted to its definitive host: it can reside in the dog gut for long periods without causing any apparent damage; the dog, in turn, usually develops an immune response that has little effect on the parasite [2], [3]. Specific anatomical structures allow a very close contact at the canid-worm interface; indeed, the intimacy of this contact has led *E. granulosus* to be regarded as both a tissue and a luminal parasite [4]. At the onset of infection, freshly evaginated protoscoleces attach to the mucosa at the base of a crypt of Lieberkhün by means of suckers, with a rostellum pushed deeply into the crypt (occasionally, even reaching the lamina propria). The apical end of the scolex contains the rostellar gland, whose secretion is thought to be important for protoscolex development [5]. The specific molecular mechanisms by which larval worms establish a successful infection in the hostile environment of the dog duodenum are, however, largely unknown.

With the aim of identifying molecules participating in the *E. granulosus*–dog cross-talk, we surveyed the genes expressed by protoscoleces and protoscoleces treated with pepsin at pH 2. Because the larval worms are naturally exposed to these signals immediately after being ingested by the dog, the rationale was that pepsin/H^+^ treatment would induce the expression of relevant genes for parasite establishment in the definitive host [6]. Analysis of the larval worm transcriptome (Parkinson J, Maizels RM, Fernández C, unpublished) revealed the existence of a multigene family of Kunitz inhibitors expressed mostly in pepsin/H^+^-treated protoscoleces, suggesting that these molecules play a role at the initial phases of infection. Kunitz inhibitors are a class of serine protease inhibitors present in all metazoa, whose prototype is the bovine pancreatic inhibitor of trypsin (BPTI; family I2 of the MEROPS database [7], [8]). They are competitive inhibitors acting in a substrate-like manner, that form very stable complexes of 1∶1 stoichiometry with their target enzymes, devoid of activity [9]. Kunitz inhibitors are also frequent components of the venoms from poisonous animals (snakes [10]; sea anemones [11], [12]; cone snails [13]; spiders [14]); in such cases, they are referred to as “Kunitz-type toxins”. Interestingly, some Kunitz-type toxins display a different activity besides serine protease inhibition: they block various types of cation permeating channels. Furthermore, several examples exist of Kunitz-type toxins acting solely as channel blockers; some neurotoxins present in the venoms of mamba snakes (“dendrotoxins”), whose function is to paralyze the prey, are the best characterized example [15].

In this article, we present the relevant molecular features of the *E. granulosus* family of Kunitz-type inhibitors which, to date, includes eight members: *Eg*KU-1 to *Eg*KU-8 (*E. granulosus* Kunitz protein 1 to 8). In addition, we describe the purification to homogeneity of *Eg*KU-1 and *Eg*KU-8 from larval worms and provide evidence of the occurrence of some members of the family in protoscolex secretions. We also present the results of detailed kinetic studies of the purified inhibitors with a panel of serine proteases that highlight their functional diversity: *Eg*KU-8 is a slow tight-binding inhibitor of trypsins; whereas *Eg*KU-1 does not inhibit any of the assayed peptidases. Interestingly, molecular modeling reveals that structural elements associated with activity of α-dendrotoxin, which is a selective blocker of specific voltage-activated K^+^-channels, are also present in *Eg*KU-1. Considered globally, our results allow us to propose that the expression of this gene family is a strategy that allows *E. granulosus* to control host processes and contribute to initiate a successful infection in the dog duodenum.

## Results

### Protoscoleces express a family of diverse Kunitz inhibitors

**Figure 1:**
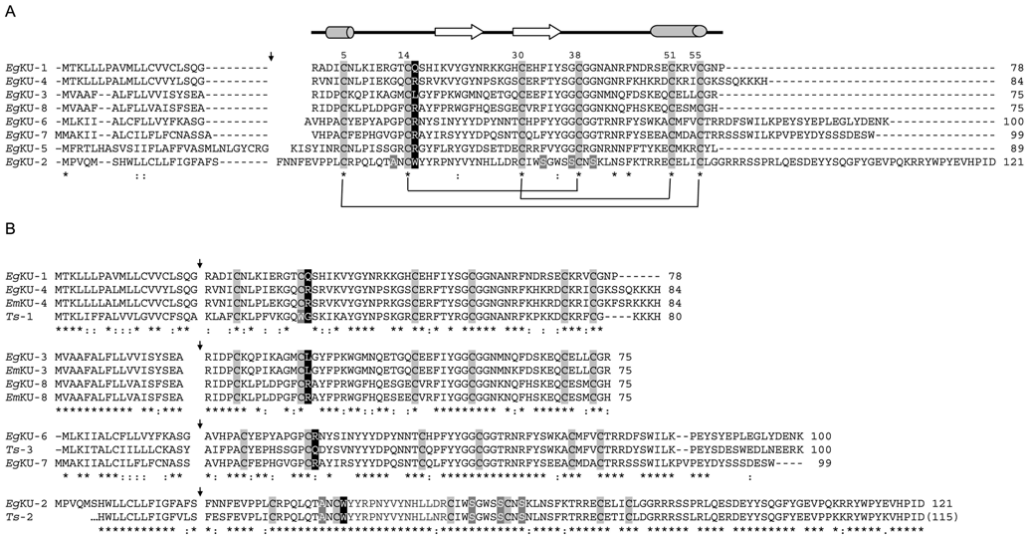
The *E. granulosus* Kunitz family and related cestode proteins. (A) Comparison of the full-length amino acid sequences of *Eg*KU-1 – *Eg*KU-8. The alignment was constructed using Clustal W2 [70] and manually refined to separate predicted signal peptides and mature proteins (the putative signal peptidase cleavage site is marked with an arrow). Conserved Cys residues are highlighted in grey and the canonical topology of disulfide bonds indicated; numbers refer to mature BPTI (58 amino acids), as well as the elements of secondary structure specified on top of the alignment to provide a global view of the domain fold. Residues present throughout are marked with (*) and conservative replacements with (:). Amino acids at position 15 (corresponding to the P1 site of serine protease inhibitors) are in white with black shading, and unusual substitutions in *Eg*KU-2 (at positions 12, 33, 37 and 40) in white with dark grey shading. (B) Comparison of *E. granulosus* and related cestode Kunitz proteins. Alignments were constructed as in (a) with the three pairs of close *E. granulosus* paralogs – *Eg*KU-1/*Eg*KU-4, *Eg*KU-3/*Eg*KU-8 and *Eg*KU-6/*Eg*KU-7 – and *Eg*KU-2, together with highly similar proteins predicted from *E. multilocularis* and *T. solium* ESTs. *Em*KU-3, *Em*KU-4 and *Em*KU-8 correspond, respectively, to XvEMa04137, XvMa03312 and XvMa16368 in Full Echinococcus [19]; *Ts*-1, *Ts*-2 and *Ts*-3 were deduced from sequences EL763407, EL746785 and EL743839 in dbEST (refer to Table S1 for further details). Unusual substitutions in the Kunitz domains of *Eg*KU-2 (conserved in *Ts*-2) are indicated as in (A); the substitution of the second conserved Cys in *Ts*-1 is similarly marked.

In the context of a strategy to identify molecules participating in the host-parasite cross-talk in hydatid infections, we undertook an EST-based transcriptome analysis of *E. granulosus* larval stages [6]. A major feature of the protoscolex transcriptome was the identification of seven members of the Kunitz family of inhibitors that we named *Eg*KU-1-*Eg*KU-7 (Figure 1A and Table S1). Interestingly, 5 of these transcripts were associated with the pepsin/H^+^-treated parasites; furthermore, ESTs from cDNAs encoding Kunitz inhibitors represented about 1% of the total derived from the treated protoscolex cDNA library (16 out of 1500), but only 0.1% (2 out of 1500) from the library of untreated parasites. Notably, two transcripts (*Eg*KU-1 and *Eg*KU-2) were among the products with highest representation in the treated protoscolex library (see Table S1).

It is predicted from sequence analyses that these proteins are secreted and the corresponding mature peptides contain a single “Kunitz domain”: about 50 amino acids forming a compact α + β structure (two short segments of α-helix located at the N and C-terminal ends of the domain + two β strands), cross-linked by three disulfide bonds between the conserved Cys residues, arranged in the canonical topology 1∶6, 2∶4 and 3∶5. As usual among members of the Kunitz/I2 family, similarity of the *E. granulosus* proteins is higher towards the C-terminal half of the domain, whereas the antiproteinase site (the P_1_ position, 15 in Figure 1A, and neighboring residues - notation of Schetcher and Berger, [16]) is within its most variable region. While all showing the architecture of a signal peptide followed by a single Kunitz domain, an extended C-terminal region is seen in some proteins (*Eg*KU-2, *Eg*KU-6 and *Eg*KU-7). In addition, they differ in isoelectric point: *Eg*KU-3 and *Eg*KU-7 are acidic, *Eg*KU-6 neutral; the remaining basic or very basic. Perhaps most significantly, the homologs differ in the residue present at the reactive P1 site; consequently, if behaving as serine protease inhibitors, diverse specificities could be expected: an Arg in P_1_ (as in *Eg*KU-4 – *Eg*KU-8) is associated with activity towards trypsin-like peptidases; Trp (*Eg*KU-2) and Leu (*Eg*KU-3) towards chymotrypsin-like enzymes; whereas Gln (*Eg*KU-1) -although rare at P_1_ sites- is compatible with activity towards various serine proteases [17], [18] (Figure 1A and Table S1).

A detailed inspection of the sequences indicated another interesting feature of the family, namely, that *Eg*KU-1/*Eg*KU-4 and *Eg*KU-6/*Eg*KU-7 represent two pairs of close paralogs, which possess highly similar predicted mature proteins and, in the case of *Eg*KU-1/*Eg*KU-4, almost identical signal peptides (Figure 1A).

When the Kunitz domains of *Eg*KU-1 to *Eg*KU-7 were compared with protein domain databases, good similarity was observed with homologous domains in other proteins (present in either single or multi-, homo as well as hetero- domain proteins) with high levels of identity, except for *Eg*KU-2 (Table S1). Furthermore, the Gly12, Phe33, Gly37 and Gly40 residues, which are conserved in the whole Kunitz/I2 family, are also present in the *E. granulosus* proteins, except in *Eg*KU-2 (where they are substituted by Ala12 and Ser at the other positions, see Figure 1A). These observations highlighted that *Eg*KU-2 is a rather atypical Kunitz protein.

Because substantial sequence information is now available from platyhelminth species previously under-represented in databases, appropriate searches were carried out using *Eg*KU-1 to *Eg*KU-7 as queries against EST databases (dbEST and also databases associated with specific sequencing projects). This led to the identification of highly similar sequences among other platyhelminths with likely orthologs of *Eg*KU-3 and *Eg*KU-4, and of *Eg*KU-2 respectively, among ESTs from *E. multilocularis* [19] and *Taenia solium* [20]. In the case of *E. multilocularis*, for which full-length cDNAs are available, the corresponding “*Em*KU-3” and “*Em*KU-4” predicted proteins were more than 90% identical to *Eg*KU-3 and *Eg*KU-4 over the whole sequence, including the signal peptide. In the case of *T. solium* “*Ts*KU-2”, identity was about 85% and also extended to the available partial signal peptide sequence. Moreover, this analysis allowed cDNAs encoding additional Kunitz inhibitors similar to the *E. granulosus* proteins to be identified. Indeed, a second cDNA related to *Eg*KU-3 (70% identity) was found to be present in *E. multilocularis*; and a close homolog of *Eg*KU-6 (75% identity) as well as several cDNAs related to *Eg*KU-4 (about 60% identity) in *T. solium*. Finally, sequences bearing significant similarity with the Kunitz domain of *Eg*KU-5 were recognized among ESTs from the free-living planarians *Dugesia ryukyvensis* and *Schmidtea mediterranea* (up to 60% identity) (Figure 1B and Table S1).

Sequence alignment of the newly-identified proteins highlighted the striking (though not unprecedented) level of identity between putative orthologs of both *Echinococcus* species, qualitatively similar at the nucleotide level. In addition, it revealed that the two *E. multilocularis* molecules similar to *Eg*KU-3 would constitute a pair of close paralogs, as previously noted for *Eg*KU-1/*Eg*KU-4 and *Eg*KU-6/*Eg*KU-7. We thus hypothesized that the second member of the pair would also be expressed in *E. granulosus* and attempted to isolate the corresponding full-coding cDNA with a set of oligonucleotide primers designed on the basis of the *E. multilocularis* sequence. RT-PCR using RNA from pepsin/H^+^ treated protoscoleces yielded a product migrating as a single band in agarose-gel electrophoresis. Sequencing of the cloned PCR product revealed an open reading frame of 228 nt encoding a 75 amino acids polypeptide, differing from the *E. multilocularis* amino acid sequence in a single residue (position 46 was Glu in *E. granulosus* and Gly in *E. multilocularis*; identity at the nucleotide level was also very high, 225/228). This new member of the *E. granulosus* Kunitz family was named *Eg*KU-8 (Figure 1A and Table S1).

Phylogenetic analysis of the Kunitz domains from *E. granulosus* and related platyhelminth sequences (see Table S1) confirmed the relatedness among sequences from different species and, also, that the family includes three pairs of close paralogs that would be the products of recent gene duplications. In the case of the “KU-3/KU-8” pair, two genes were already present in the common ancestor of the two *Echinococcus* species (Figure S1). This analysis also emphasized that *Eg*KU-2 is an atypical Kunitz protein; interestingly, the same unusual substitutions of conserved residues (Gly12Ala, Phe33Ser, Gly37Ser and Gly40Ser) were observed in the putative ortholog identified in *T. solium* (Figure 1B).

### Kunitz inhibitors may be purified from protoscoleces and detected in their secretions

**Figure 2:**
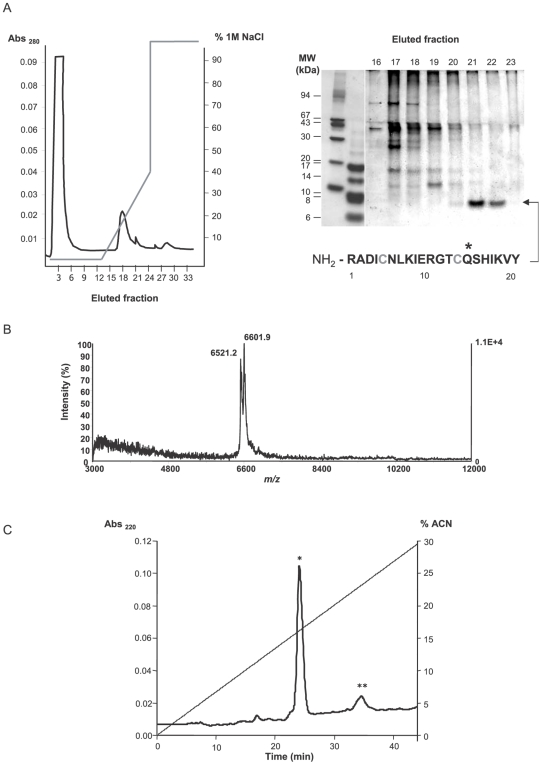
Purification of *Eg*KU-1 and *Eg*KU-8 from a protoscolex extract. (A) Fractionation of cationic protoscolex proteins. *Left panel*, Chromatography profile (MonoS column; pH 7); elution was with increasing NaCl concentrations. *Right panel*, Tricine SDS-PAGE of eluted fractions under non-reducing conditions; the gel was Coomassie-stained. N-terminal sequencing of the 7 kDa band identified *Eg*KU-1 in fractions 21 and 22 (see Figure 1A). Conserved Cys are in grey and the putative P1 site marked with an asterisk. (B) MALDI-TOF MS of *Eg*KU-1containing fractions. The peak at m/z 6601.9 matched the MH^+^ value predicted for *Eg*KU-1 (6600.5 Da); similarly, the 6521.2 signal indicated that the fractions also contained *Eg*KU-8 (MH^+^  = 6520.4 Da). MW estimation was from cDNA predicted sequences, considering that conserved Cys form disulfide bonds (see Table S1). (C) Separation of *Eg*KU-1 and *Eg*KU-8 by rpHPLC. A pool of the ion exchange fractions containing the 7 kDa band was loaded onto a C8 column. A major and a minor peak were eluted with acetonitrile (ACN) in 0.07% trifluoroacetic acid, at about 16% (*) and 24% (**) ACN. By MALDI-TOF MS, each peak was found to contain one predominant component corresponding, respectively, to *Eg*KU-1 and *Eg*KU-8, as specified in (B).

As part of an independent strategy aimed at isolating positively charged molecules from *E. granulosus* larval worms, a soluble extract was fractionated by cationic exchange chromatography at pH 7. Column elution with a linear NaCl gradient yielded two peaks that were analyzed by non-reducing Tricine-SDS-PAGE. Interestingly, despite the fact that the starting material was a crude preparation, the profile of the fractions corresponding to the minor peak (fractions 20 to 23 in Figure 2A) was extremely simple: they contained a major band of approximately 7 kDa. This band was submitted to N-terminal sequencing, and the 21 determined residues found to correspond to the mature form of *Eg*KU-1, the most abundant Kunitz inhibitor identified in the protoscolex transcriptome (see Figures 1A and 2A).

Although the N-terminal amino acids were unambiguously assigned and only one signal was obtained in each sequencing cycle, further analysis of the fractions containing *Eg*KU-1 by mass-spectrometry revealed two peaks (Figure 2B) of m/z 6601.9 and 6521.2, matching the predicted monocharged molecular ion mass (MH^+^) for the mature forms of *Eg*KU-1 (6600.5 Da) and *Eg*KU-8 (6520.4 Da), respectively (see Table S1; note that both inhibitors would be positively charged at pH 7). The two components were separated by rpHPLC, as confirmed by MALDI-TOF MS (Figure 2C). Peptide finger-printing of the component recovered in the second rpHPLC peak (whose molecular mass matched the MW of *Eg*KU-8) allowed confirmation of its identity and its unequivocal assignment to *Eg*KU-8 (Figure S2). *Eg*KU-1 and *Eg*KU-8 were thus purified to homogeneity from a protoscolex extract using a combination of cation exchange and reverse phase chromatography.

**Figure 3:**
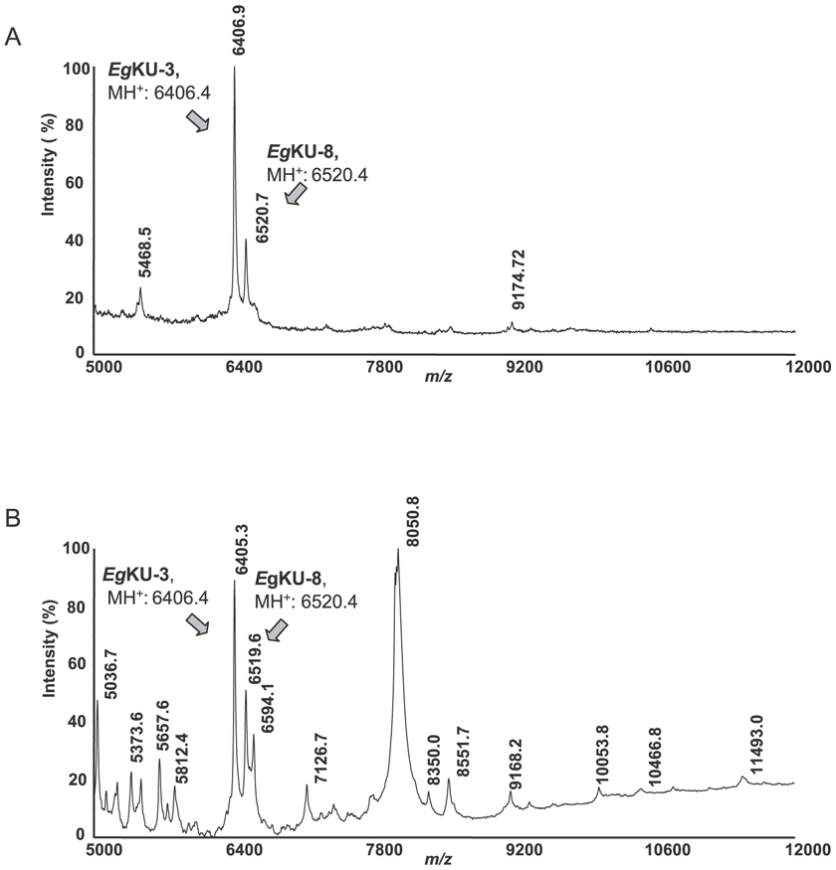
Detection of members of the Kunitz family in protoscolex secretions. Analysis by MALDI-TOF MS of supernatants from short-term cultures of (A) untreated and (B) pepsin/H^+^-treated protoscoleces. The spectra highlight the different complexity of the two samples. Signals whose m/z values could derive from the presence of *E. granulosus* Kunitz inhibitors are indicated, together with the MH^+^ predicted for each protein based on translation of the corresponding cDNA, considering that the six conserved Cys residues form disulfide bonds (seeTable S1).

To approach the question of whether Kunitz inhibitors are, indeed, secreted to the parasite-host interface, we analyzed the supernatants from cultured protoscoleces by mass spectrometry. Aliquots from freshly isolated parasites were either left untreated or treated with pepsin/H^+^ prior to the culture. Figure 3 shows representative MALDI-TOF MS profiles (5000 – 12000 Da) of supernatants from short-term (3 h) cultures of the larval worms. Two major peaks of m/z 6406.9 and 6520.7, matching the predicted MH^+^ value for *Eg*KU-3 (6406.4 Da) and *Eg*KU-8 (6520.4 Da), respectively (Figure 3A), were observed in the supernatant from untreated worms. The secretions from pepsin/H^+^-treated protoscoleces were considerably more complex and included signals of m/z 6405.3 and 6519.6, also consistent with the presence of *Eg*KU-3 and *Eg*KU-8 (Figure 3B). A peak of m/z 6594.1 was detected as well; while close, this does not accurately match the MH^+^ predicted for *Eg*KU-1 (within about 1 Da as was the case for the other molecules and also for *Eg*KU-1 in the ion exchange eluate, Figure 2B). Although the identity of the peaks needs further confirmation, the results indicate that members of the Kunitz family would be present in protoscolex secretions at the onset of infection. It is worth noting that *Eg*KU-3 is an acidic protein that would be negatively charged at pH 7; thus, even if it had been present in the starting protoscolex extract, it would not have bound to the cation exchange column used for the isolation of *Eg*KU-1 and *Eg*KU-8.

### *Eg*KU-8 is a high affinity trypsin inhibitor whereas no inhibition of serine protease activity was detected with *Eg*KU-1

A preliminary screening (not shown) of serine protease inhibitory activity was carried out with purified native *Eg*KU-1 and *Eg*KU-8. Taking into account the usual inhibition profiles of proteins from the Kunitz family, we assayed pancreatic enzymes (bovine cationic trypsin and chymotrypsin A; and porcine elastase) and serine proteases of the coagulation cascade.

*Eg*KU-8 displayed dose-dependent inhibitory activities against trypsin and chymotrypsin, whereas no inhibition of elastase was detected, even at high concentrations of the parasite protein. As expected for Kunitz inhibitors which are extremely stable proteins [21], [22], the activity was heat-resistant: around 80% and 65% of the inhibitory capacity towards trypsin and chymotrypsin respectively, was retained after 20 min at 100°C. Activity towards serine proteases of the coagulation cascade was tested through measurement of prothrombin time and partial thromboplastin time; two functional assays which are highly sensitive for factors X, VII and II, and factors XII, XI, X, IX and II, correspondingly. No increase of either time was observed using normal human plasma, indicating that *Eg*KU-8 did not inhibit these enzymes.

**Figure 4:**
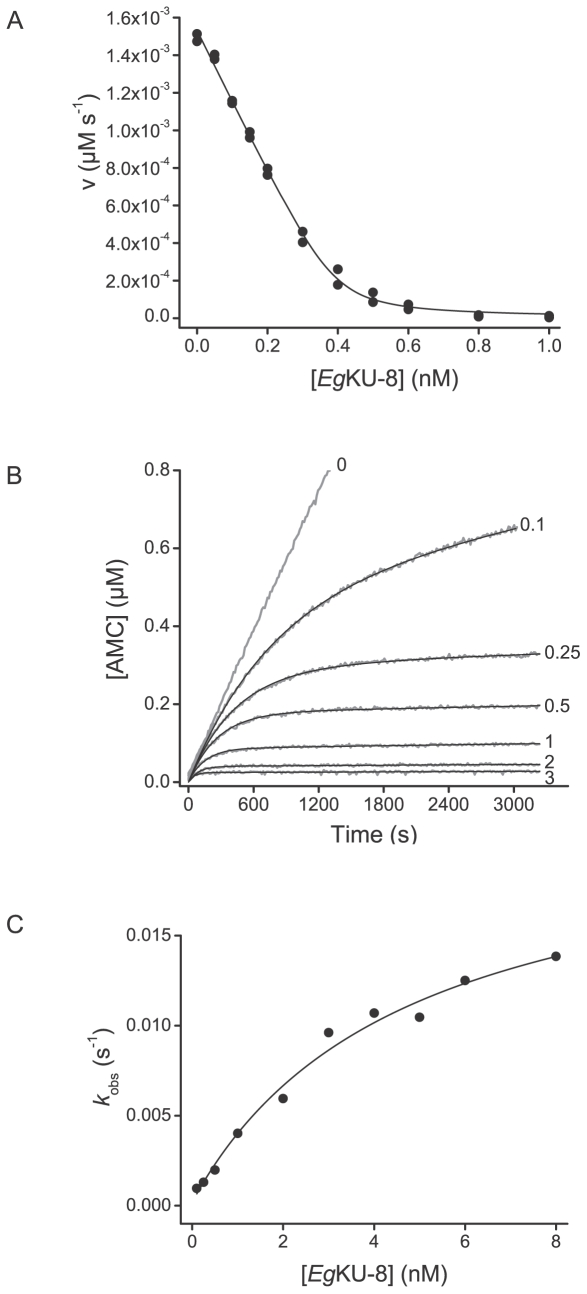
Inhibition studies with *Eg*KU-8: results for canine anionic trypsin. (A) Inhibition of canine anionic trypsin. The enzyme (0.30 nM) was preincubated for 15 min with *Eg*KU-8 (0.05–1.0 nM) and mixed with substrate (N-*t*-BOC-Ile-Glu-Gly-Arg-AMC, 5 µM) in 50 mM Tris-HCl, pH 8.0, 0.01% Triton X-100, at 37°C. *K* ^*^_I, app_ values at equilibrium were determined from the remaining activity using equation (1) for tight binding inhibitors as described in Materials and Methods. The solid line represents the best fit to this equation. (B) Progress curves for the inhibition of canine anionic trypsin. The enzyme (0.05 nM) was added to reaction mixtures containing the substrate (N-*t*-BOC-Ile-Glu-Gly-Arg-AMC, 5 µM) and increasing concentrations of *Eg*KU-8 (0, 0.1, 0.25, 0.5, 1, 2, and 3 nM, gray traces) in 50 mM Tris-HCl, pH 8.0, 0.01% Triton X-100, at 37°C. The black traces represent the best fits to equation 3, from which *k*_obs_ were obtained. (C) Dependence of *k*_obs_ on the concentration of inhibitor for canine anionic trypsin. The enzyme was added to reaction mixtures containing the substrate (N-*t*-BOC-Ile-Glu-Gly-Arg-AMC, 5 µM) and increasing concentration of *Eg*KU-8 in 50 mM Tris-HCl, pH 8.0, 0.01% Triton X-100, at 37°C. The enzyme concentrations were: 0.05 nM for 0.1–1 nM of *Eg*KU-8, 0.2 nM for 1–5 nM of *Eg*KU-8, and 0.6 nM for 5–8 nM of *Eg*KU-8. *k*_obs_ values were obtained from time course experiments according to equation 3 and correspond to the average of at least two independent determinations. The black trace represents the best fit to equation (4) in agreement with scheme 1.

In view of the behavior of *Eg*KU-8 with bovine trypsin and chymotrypsin, and assuming that the host digestive enzymes could be physiological targets of the parasite molecule, we next analyzed its activity against trypsin and chymotrypsin purified from dog pancreas, *i. e.* anionic and cationic trypsins and chymotrypsin B (chymotrypsin A is absent from dogs, see S01.001 at MEROPS - http://merops.sanger.ac.uk). In parallel, we also assayed the bovine enzymes (*i. e.* cationic trypsin and chymotrypsin A). To obtain global inhibition constants (*K*_I_^*^) for *Eg*KU-8, data of *v*_i_ *versus* [I] were fit to equation (1). Representative results for canine anionic trypsin are shown in Figure 4A, and the values of *K*_I_ ^*^ for each enzyme in Table 1. *Eg*KU-8 behaved as a very efficient inhibitor of both canine trypsins, with *K*_I_^*^ of 17±4 and 22±8 pM for the cationic and anionic enzymes, respectively (Table 1). It also inhibited bovine trypsin although to a lower extent than the canine counterparts, since *K*_I_^*^ was ∼3-fold higher. In contrast, no appreciable inhibition of chymotrypsin B was detected, while the *K*_I_^*^ obtained for chymotrypsin A was in the nanomolar range, two orders of magnitude higher than the *K*_I_^*^ for trypsins. Results of *Eg*KU-8 with chymotrypsins reproduced those obtained with BPTI, which was also found to inhibit chymotrypsin A but not the B isoforms of the protease, from either bovine [23] or from canine (our unpublished observations) origin.

**Table 1:** Inhibition constants (*K*_I_^*^) of *Eg*KU-8 acting on digestive serine proteases.

Target enzyme	*K*_I_^*^ (pM)^a^
Bovine	
Cationic trypsin	60±13
Chymotrypsin A	2050±170
Canine	
Anionic trypsin	17±4
Cationic trypsin	22±8
Chymotrypsin B	NI^b^

a *K*_I_^*^, the global equilibrium dissociation constants, were calculated from inhibition assays (see Figure 4A) according to equation 1 for tight-binding inhibitors and minimally corrected for the effect of substrate concentration according to equation 2. Values correspond to averages of independent measurements ± the standard error (n≥3).

b NI, not inhibited.

The progress curves for the inhibition, as shown for anionic trypsin in Figure 4B, indicated that, in the presence of *Eg*KU-8, the rate of substrate hydrolysis reached the inhibited steady-state rate in a time scale of minutes, suggesting that the formation of the enzyme-inhibitor complex is a slow process and that *Eg*KU-8 is a slow-binding inhibitor as defined by Morrison [24]. The interaction of *Eg*KU-8 with all three trypsins was reversible, since progress curves reached appreciable slopes even at higher than stoichiometric inhibitor concentrations. This is the behavior expected for Kunitz-type inhibitors [9].

Similarly, plots of the apparent rate constant (*k*_obs_) *versus Eg*KU-8 concentration were hyperbolical (Figure 4C), in accordance with the mechanism shown in Scheme 1. The kinetic constants of *Eg*KU-8 binding to the bovine and canine trypsins obtained from analyses of the progress curves are shown in Table 2. The values of *k*_2_ and *k_−2_* were in the order of 10^−2^ and 10^−4^ s^−1^, consistent with the fact that *Eg*KU-8 behaved as a slow inhibitor. *K*_I_, the equilibrium dissociation constant of the initial loose complex, was in the nanomolar range. Remarkably, *K*_I_ was 2–3-fold higher for bovine trypsin than for the canine enzymes. The values of *k*_2_/*K*_I_, the apparent second order rate constants for complex formation (*k*_on_), were in the order of 10^6^ M^−1^ s^−1^. Although slight differences in *k*_2_ and *K*_I_ were observed between the canine trypsins, the ratio *k*_2_/*K*_I_ was similar for both isoforms. In turn, as *K*_I_ was 2–3-fold higher for the bovine trypsin compared to the canine enzymes, the ratio *k*_2_/*K*_I_ was accordingly lower. The fact that *K*_I_ was higher for the bovine than for both canine isoforms may explain the differences observed in *K*_I_^*^, according to equation 6. The values of *K*_I_^*^, calculated from the kinetic constants (Table 2), compared very well with the values obtained through the fit of steady-state data to the Morrison equation (Table 1).

**Table 2:** Inhibitory kinetics of *Eg*KU-8 on bovine and canine trypsins.

Kinetic constant	Cationic bovine trypsin	Anionic canine trypsin	Cationic canine trypsin
*k*_2_×10^−2^ (s^−1^)^a^	2.6±0.4	2.8±0.6	1.5±0.1
*K*_I_ (nM)^a^	10.2±3.4	4.3±0.5	2.9±1.0
*k*_2_/*K*_I_×10^6^ (M^−1^ s^−1^)^a^	2.7±0.5	7.0±1.2	6.0±2.0
*k*_−2_×10^−4^ (s^−1^)^b^	2.3±0.3^c^	1.1±0.7	1.1±0.5
*K*_I_^*^ (pM)^d^	ND^e^	16±3	21±6

a *k*_2_, *K*_I_ and *k*_2_/*K*_I_ were calculated from time course experiments (see Figures 4B and 4C) according to the fit to equation 4 of *k*_obs_ versus [I] plots. Values are averages of independent measurements ± the standard error (n≥2).

b *k*_−2_ were calculated from time course experiments according to equation 5. Values are averages of independent measurements ± the standard deviation (n = 15).

c Calculated from equation 6 using the value of *K*_I_^*^ determined in steady state inhibition experiments (Table 1). The value is the average of independent measurements ± the standard error (n = 2).

d *K*_I_^*^ were calculated from equation 6 using the values of *k*_2_, *K*_I_ and *k*_−2_ obtained from time course experiments. The values are averages of independent measurements ± the standard error (n≥3).

e ND, not determined.

In sharp contrast with *Eg*KU-8, *Eg*KU-1 did not inhibit any of the assayed proteases (*i. e.* trypsin, chymotrypsin, elastase and serine proteases from the coagulation cascade).

## Discussion

A central theme of parasite adaptation is the study of host-parasite interfaces and the interactions between molecules from both organisms that, ultimately, underlie a successful parasitism. The products secreted by infective stages are especially relevant in this context: parasite establishment relies, to a great extent, on the capacity of these molecules to give rise to fast, high affinity interactions with their host counterparts. Such finely tuned host-parasite cross-talk is the result of a co-evolutionary process where each molecular partner has been selected in response to changes in the other (see, for example, [25]). Serine protease inhibitors have been recognized as key components of parasite secretions and have been implicated in parasite survival through their capacity to inhibit host enzymes, either normally present in the microenvironment and/or secreted by immune effector cells (see, for example, [26]).

In the present work, we describe a family of eight Kunitz-type inhibitors from *E. granulosus* protoscoleces, which is preferentially expressed after exposure to signals such as those encountered in their definitive host. In addition, we have provided evidence that some of these proteins are synthesized prior to infection of dogs (*Eg*KU-1 and *Eg*KU-8) and secreted by the larval worms (*Eg*KU-3 and *Eg*KU-8). A detailed analysis of the time course of the synthesis and secretion of all the members of the family as well as of the signals regulating these processes is ongoing; nevertheless, the results presented herein point to a role of these molecules at the onset of echinococcosis. The most notable features of the family at the molecular level are its diversity and the existence of several pairs of close paralogs (Figure 1 and Table S1). These two features are consistent with an accelerated evolution of the family, and it is to be expected that the targets of inhibition -as counterparts- reproduce a similar pattern of diversity. One member, *Eg*KU-2, is only distantly related to the rest (<25% pair-wise identity); the other members have 35% to 45% pair-wise identities, rising to >70% between close paralogs (71% between *Eg*KU-1/*Eg*KU-4; 76% for *Eg*KU-6/*Eg*KU-7; and 82% for *Eg*KU-3/*Eg*KU-8). Consistent with the idea of accelerated evolution to generate functional diversity is the fact that, in two pairs of paralogs (*Eg*KU-1/*Eg*KU-4 and *Eg*KU-3/*Eg*KU-8), identity in the signal peptides is higher than in the Kunitz domains.

Interestingly, our exhaustive search for platyhelminth Kunitz inhibitors in EST databases indicated that, among parasites, the expression of “families” of these proteins would be a distinctive trait of cestodes. Indeed, at least three putative orthologs of the *E. granulosus* molecules were identified in each of the other two medically important cestodes, *E. multilocularis* and *T. solium*; whereas no cDNAs coding for proteins with a similar molecular architecture (an N-terminal signal peptide followed by a single Kunitz domain) were spotted in the large collection of trematode ESTs, which includes extensive surveys from the transcriptomes of several species (in particular, from practically all life-cycle stages of *Schistosoma mansoni* [27], and several from *S. japonicum* [28]). Although the transcriptomes of the “activated” infective stages from other cestodes have not yet been analyzed, it is tempting to speculate that the secretion of Kunitz inhibitors is an evolved strategy to establish in the duodenum of their definitive hosts. As could be expected from the extremely high similarity of both Echinococcus, putative orthologs of *Eg*KU-1 – *Eg*KU-8 were identified when searching the recently assembled *E. multilocularis* genome (at the Sanger Institute BLAST server: http://www.sanger.ac.uk/cgi-bin/blast/submitblast/Echinococcus). This search also highlighted that the “cestode Kunitz family” likely includes more than eight members.

cDNAs encoding Kunitz inhibitors were also identified among ESTs from the free-living planarians. A search of the *S. mediterranea* genome database (SmedGD, [29]) indicated that this organism has quite a large set of predicted single domain Kunitz proteins (at least 20) of which 14 are known to be transcribed from EST data. The fact that these molecules appear to be present in planarians and cestodes but virtually absent from trematodes highlights that the evolution of the Kunitz family is lineage specific within platyhelminths, of special interest if, as we suggest, it would be related to the parasitic way of life of cestodes.

In the case of nematodes, the other phylum of helminths, which also includes parasites and free-living organisms, a search of NEMBASE [30] for proteins predicted to contain Kunitz domains identified more than 100 EST clusters in species from all nematode clades, including the free-living Rhabditoidea. A majority of these domains (all of them in the case of *Caenorhabditis elegans* [8]) appear to be present in a diverse group of molecules containing Kunitz and other domains, with one to twelve Kunitz domains in the same protein [31], [32]. However, it is noteworthy that about 25 clusters corresponded to putative Kunitz inhibitors (*i. e.* proteins with a signal peptide and a single Kunitz domain) from animal and plant parasites (clades V and VI, respectively), with several clusters derived from just a few species. Thus, the presence of “families” of Kunitz inhibitors would also be associated with a parasitic way of life in some nematodes. Interestingly, a second family of canonical serine protease inhibitors is expressed by these organisms (Ascaris/I8 family at the MEROPS database, see [26]); a number of parasitic nematodes (such as *Ancylostoma* spp.) express members from each of the two families (I8 [33] as well as I2 [34]).

The other major finding from our work derives from functional data showing that *Eg*KU-1 could inhibit neither bovine trypsin nor chymotrypsin, whereas *Eg*KU-8 behaved as a powerful inhibitor of both enzymes (Figure 4, Tables 1 and 2). The results with *Eg*KU-8 complement detailed studies of the interaction of BPTI and its mutants with classical serine proteases [18], [35], which have elucidated the structure-function relationship and broad specificity of the Kunitz family. The antiprotease site is primarily formed by the canonical loop stabilized by the Cys14-Cys38 disulfide bond: about 8 amino acids surrounding the P_1_ residue (residues P_4_-P_4_′, positions 12–19 in BPTI) are in direct contact with the protease. Major players in this interaction are the residue in P_1_, which is highly complementary to the active site of the enzyme (S_1_ pocket) and establishes 50% of the interactions; and also those in P_3_, P_1_′, P_2_′ and P_4_′. In other molecules, like the first Kunitz domain of human tissue factor pathway inhibitor-2 [TFPI-2 (1)], residues in P_6_ and P_5_′ are also important determinants of the specificity of inhibition [36].

**Figure 5:**
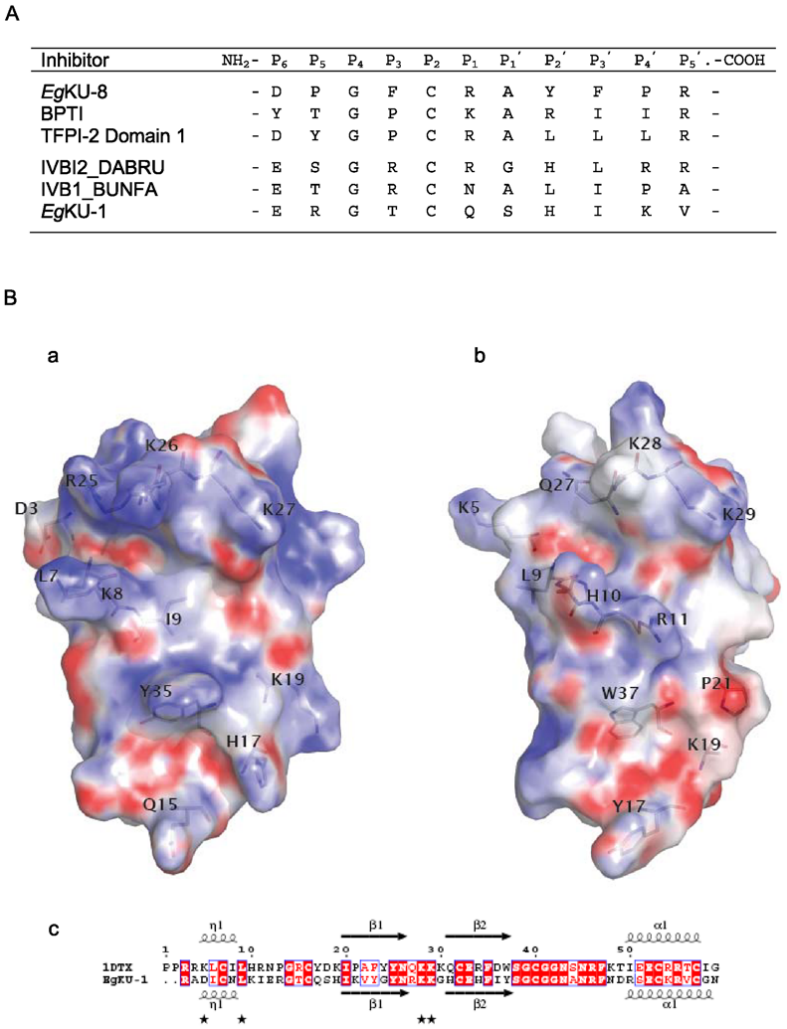
Structure-activity analysis of *Eg*KU-1 and *Eg*KU-8. (A) Amino acid sequences surrounding the P_1_ reactive site residue of selected Kunitz-type inhibitors. Residues at positions 10 to 20 of the *E. granulosus* proteins are compared with equivalent amino acids from BPTI (sp P00974); TFPI-2 Domain 1 (sp P48307); and two proteins from snake venoms – from *Daboia russelli* (IVBI2_DABRU;sp P00990) and *Bungarus fasciatus* (IVB1_BUNFA; sp P25660). (B) Electrostatic surface potentials of: (a) *Eg*KU-1 homology model and (b) α-dendrotoxin crystal structure (PDB access code: 1dtx, chain A). Homology modeling was done with Modeller [52] using thirteen structures adopting the Kunitz fold (with the following PDB access codes and chains: nine protease inhibitors -1aapA, 1tfxC, 1btiA, 3btgI, 1co7I, 1zr0B, 1jc6A, 1kntA, 1bikA, 1demA; three channel blockers -1demA, 1dtxA, 1dtkA; one domain from collagen -1bcrI). Structurally equivalent positions in EgKU-1 and α-dendrotoxin are labeled, with 2 positions shift in the primary sequence. Molecular surfaces are color-coded from positive blue [+300 mV] to negative red [−300 mV], through a neutral white. Electrostatic calculations were performed using the Adaptive Poisson-Boltzmann Solver [71]. To illustrate the sequence relationship between *Eg*KU-1 and its best template, a pair-wise alignment of *Eg*KU-1 and α-dendrotoxin (45% sequence identity) (c) was produced with ESPript [72]. Identical residues are shown in bold white on a red background; conservative substitutions are indicated in light red on a white background. Some additional positions discussed in the text (1DTX: K5, L9, K28, K29; *Eg*KU-1: L7, K26, K27) are highlighted by asterisks.

The activity of *Eg*KU-8 as a strong tight-binding inhibitor of all three assayed trypsins and a less potent inhibitor of chymotrypsin A (Tables 1 and 2) is consistent with the residues of its canonical loop, similar to those in BPTI and, even more, in TFPI-2 (1), a strong inhibitor of trypsin and plasmin [36] (Figure 5A). This observation indicates that other trypsin-like proteases as well as trypsin, present in the protoscolex establishment scenario, could be the molecular counterparts of *Eg*KU-8. The interaction between *Eg*KU-8 and trypsins is slow, tight and reversible, resembling other Kunitz/I2 inhibitors with their target enzymes [21], [37], [38]. The stability of the corresponding inhibitor-enzyme complexes is reflected by the small rate constants for their dissociation, in the order of 10^−4^ s^−1^. The values of *k*_2_/*K*_I_, the apparent second order rate constants for complex formation (*k*_on_), in the order of 10^6^ M^−1^ s^−1^, are in good agreement with reports for other members of the family, including BPTI with bovine trypsin [39]. In turn, the values of *k_−_*_2_, 10^−4^ s^−1^, are several orders faster than those reported for BPTI (10^−8^ s^−1^ with bovine trypsin [39]).

The fact that the inhibition constants for canine trypsins were three-fold lower than the value for the bovine enzyme (Tables 1 and 2) is especially interesting in the context of this work. In principle, it is possible to consider it as an indication that the dog enzymes could be physiological targets of *Eg*KU-8. However, the result should be interpreted with caution because a similar pattern was observed in the values of *K*_M_ for the substrate used in the assays (see Materials and Methods) and comparable values were reported with another substrate [40], [41]. In view of these observations, we looked for variations in amino acids known to be critical for the interaction with substrates and inhibitors in the three enzymes. The S_1_ trypsin binding site is formed by residues 189–195 (loop 1) and 214–220 (loop 2) (chymotrypsin numbering [42]). The three proteins are identical over these loops except for the residue at position 217, which is Ser in the bovine, Ala in the cationic and Tyr in the anionic canine enzymes. Interestingly, the anionic trypsin from Atlantic salmon also has a Tyr217, and the presence of this residue was used to explain discrepancies in the behavior of the salmon and bovine trypsins, similar to the ones arising from our data [43], [44], [45]. The differences were considered to derive from variations in the network of hydrogen bonds at the S_1_ pocket of the respective enzyme-substrate/inhibitor complexes: direct hydrogen bonds are formed with the salmon enzyme [46], which are not observed in the complexes with bovine trypsin (instead, they are water mediated due to the Ser217 [47]). The differences we found could be similarly explained.

Regarding the results with *Eg*KU-1, it is not clear why it did not inhibit the assayed peptidases. Comparison of its putative antiprotease loop with positions P_6_-P_5_′ of known inhibitors showed that analogous amino acids are present in active molecules: the chymotrypsin inhibitor from *Bungarus fasciatus* has Asn in P_1_ and is similar over the P-side of the loop; the trypsin inhibitor from *Daboia russelli* bears equivalent residues in P_2_′ and P_4_′ (His and Arg) (Figure 5A). Thus, the occurrence of these amino acids does not explain, by itself, the lack of antiprotease activity of *Eg*KU-1. It is worth noting that it has also been complex to explain why other Kunitz domains whose structures have been thoroughly analyzed are not active against proteases [48], [49]. Interestingly, the inactive Kunitz domain of human collagen VI was rendered as active as BPTI by mutating two amino acids from its antiprotease loop and one from the hydrophobic core of the domain [50], reinforcing the view that target recognition in the Kunitz family relies on the conformation of the chain segment to which the interactive side-chains are attached [51]. However, given the known high conformational flexibility of the antiprotease loop, the effects are subtle and often hard to predict.

With these caveats concerning the antiprotease loop, and in order to further discern among possible functions of *Eg*KU-1, we attempted to get an unbiased structural insight. For this, the best templates, as defined by sequence identity criteria, were retrieved from the Protein Data Bank. Next, the Modeller program [52] was simultaneously fed with spatial restraints coming from thirteen functionally diverse structure templates (nine protease inhibitors, three cation channel blockers and the above mentioned Kunitz domain from human collagen VI). Interestingly, the best overall template was found to be α-dendrotoxin, the extensively characterized blocker of specific voltage-activated K^+^-channels [15]. We subsequently intended to check whether the structural elements associated with channel-blocking activity were also present in the reached consensus model of *Eg*KU-1. Although not so clearly delineated as the antiprotease site, the channel-blocking site of α-dendrotoxin and related toxins is formed by residues from the N-terminus and the β-turn region of the Kunitz domain, brought close to each other by the conserved Cys5-Cys55 bond. Key residues in the interaction appear to be a protruding Lys and a close hydrophobic amino acid; whereas an enrichment of basic side chains at sites forming an interface with the channel has also been a consistent finding [53], [54], [55], [56]. Analysis of the electrostatic surface potential of *Eg*KU-1 indicated that residues from its N-terminus (Lys8) and β-turn (Arg25, Lys26 and Lys27) define highly cationic protuberances that line an elongated crevasse (Figure 5B). Furthermore, a comparison with α-dendrotoxin showed that both molecules share several amino acids found to be important for activity (Leu7 and Lys27 in *Eg*KU-1 are equivalent to Leu9 and Lys29 in α-dendrotoxin); a residue comparable to the Lys5 is, though, absent from the parasite molecule. These observations would support the notion that *Eg*KU-1 is a putative cation-channel blocker. Interestingly, Leu7 and Lys27 are conserved in *Eg*KU-4 (the closest paralog of *Eg*KU-1) and also in the closest homologs from *E. multilocularis* and *T. solium* (Figure 1B).

Taken altogether, our results suggest that the secretion of Kunitz proteins is a strategy evolved by *E. granulosus* to block, through high affinity interactions, the function of host proteins (such as serine proteases and, possibly, K^+^-channels) present at the site of establishment of the larval worms. From a more general perspective, if the predicted K^+^-channel blocking activity is confirmed, this family of secreted cestode proteins would provide a striking example of protein evolution, similar to the one described in animal toxin multigene families, where natural selection has acted to diversify the coding sequences of duplicated genes, allowing the emergence not only of specific inhibitors of particular enzymes (*i. e.* paralogous genes whose products block paralogous proteins), but also of a new function associated with the same molecular scaffold [10], [57]. Taking into account that the genes coding for parasite secretions and predator toxins arise from an arms race between different organisms, it is interesting to consider that both sets of molecules display similar evolutionary patterns. Thus, the concept of “exogenome” (the part of the genome whose products are targeted exogenously [58]) coined for toxin genes, may also be applied to the genes encoding parasite secretions.

## Materials and Methods

### Analysis of protoscolex transcriptomes

Hydatid cysts (G1 genotype) from the lungs of naturally infected bovines were obtained from slaughterhouses in Uruguay. Protoscoleces were recovered under aseptic conditions, and extensively washed in phosphate-buffered saline to remove dead larval worms. They were stored at −70°C in TRIzol reagent (Invitrogen) until RNA extraction. One fraction of freshly isolated larval worms was incubated with 0.5 mg/ml of pepsin (Sigma) at pH 2 for 3 h at 37°C, prior to treatment with TRIzol. The processing of parasite materials and the construction and sequencing of full-length enriched cDNA libraries were previously described [6]. A detailed account of the bioinformatics analysis of ESTs will be provided in a separate manuscript (Parkinson J, Maizels RM, Fernández C, unpublished). In brief, sequence processing was performed using the PartiGene pipeline [59]. Low quality, vector, host (bovine), linking and poly(dA) sequences were removed from raw sequence trace data. The resulting sequences were annotated by comparison to the protein non-redundant database (UniProt - [60]) using BLAST and submitted to dbEST [61]. Sequences were subsequently collated and clustered on the basis of BLAST similarity to derive groups of sequences, which putatively derive from the same gene using the software package - CLOBB [62]. EST clusters were thus verified as originating from distinct transcripts and not from sequencing errors.

### Identification of platyhelminth cDNAs related to *Eg*KUs

The full-length sequences predicted for *Eg*KU-1 to *Eg*KU-7 were subjected to BLAST analysis for similarity to “non-human, non-rodent ESTs” (option “est_others” at the NCBI server), and *E. multilocularis* ESTs available at “Full-Echinococcus” (http://fullmal.hgc.jp/em/index.html [19]). As of August 2009, dbEST contains 24,790 ESTs from *Taenia solium*, generated from cDNA libraries of whole larva (cysticercus) and whole tapeworm; 74,915 from *Schmidtea mediterranea*, derived from various sources including libraries from juvenile and sexually mature worms of the hermaphroditic strain; and 16,350 from *Dugesia* spp. (8,988 from whole adults of *D. ryukyuensis*; and 7,362 from the head of *D. japonica*). Full-Echinococcus includes 10,966 ESTs, generated from a library of hydatid cysts developed in cotton-rats (one-third of ESTs represent host genes).

### Cloning of *Eg*KU-8 cDNA

About 1 µg of total RNA isolated from pepsin/H^+^-treated protoscoleces preserved in TRIzol was used to prepare cDNA using PowerScript reverse transcriptase (Clontech) and an oligo(dT) primer. The full coding sequence of *Eg*KU-8 was amplified from an aliquot of cDNA using forward and reverse primers designed on the basis of the sequence from the putative *E. multilocularis* ortholog [XvEMa16368 in Full-Echinococcus (http://fullmal.hgc.jp/em/index.html)]: KU8F: 5′-ATG GTT GCC GCC TTT GCG C-3′; and KU8R: 5′-AAA GCT TAC TTA GTG ACC GCA C-3′. The PCR was initiated with a touch down (5 cycles at 94°C/0.5 min, 60°C to 55°C/1 min, 75°C/1.5 min), and followed by 30 cycles at 94°C/0.5 min, 55°C/1 min, 75°C/1.5 min, using *Vent* DNA polymerase (New England Biolabs). The single product thus obtained was purified from an agarose gel, A-tailed with *Taq* DNA polymerase (Fermentas), cloned into pGEM-T-easy (Promega) and sequenced using vector primers. The corresponding cDNA sequence was admitted to GenBank with accession number: FJ031017.

### Accession numbers

*E. granulosus* sequence data reported in this manuscript is available from GenBank and the corresponding accession numbers indicated in Table S1. Accession numbers for other platyhelminth sequences are also indicated in Table S1, together with the database from which they were retrieved. Accession numbers in Swiss-Prot or TrEMBL of Kunitz domain proteins used for comparison of *Eg*KUs are specified in Table S1 and in the legend to Figure 5A. The Protein Data Bank access codes for the templates of the homology modeling of *Eg*KU-1 are provided in the legend to Figure 5B.

### Purification of native Kunitz inhibitors from protoscoleces

*Eg*KU-1 and *Eg*KU-8 were purified to homogeneity from a protoscolex lysate by cation exchange followed by reverse-phase chromatography. Freshly isolated protoscoleces were homogenized by sonication in 50 mM phosphate buffer, pH 7. The homogenate was centrifuged at 10,000×*g*, and the recovered supernatant was first subjected to FPLC on a Mono S HR 5/5 column (GE Healthcare). After loading the extract, the column was washed with 10 volumes of binding buffer (50 mM phosphate, pH 7); bound proteins were eluted with a linear NaCl gradient (0–0.4 M in 10 min, at a flow rate of 1 ml/min) in the same buffer. Elution was monitored at 280 nm; 1 ml fractions were collected and analyzed by non-reducing 15% Tricine-SDS-PAGE [63] and for serine protease inhibitory activity. Active fractions were pooled and applied to an Aquapor RP-300 (100×21 mm, Perkin Elmer) reverse phase HPLC column (rpHPLC). Bound proteins were eluted with a linear gradient of acetonitrile in 0.07% trifluoroacetic acid (0–40% in 60 min, at a flow rate of 0.4 ml/min), and monitored at 220 nm. Eluted proteins were lyophilized and dissolved in 50 mM Tris-HCl, pH 8, 0.01% Triton X-100 (v/v).

The Mono S fractions showing serine protease inhibitory activity and the rpHPLC peaks were analyzed by matrix-assisted LASER desorption ionization time-of-flight mass spectrometry (MALDI-TOF MS) using a Voyager DE-PRO instrument (Applied Biosystems). Mass spectra of whole proteins were acquired on linear mode using a matrix solution of α-cyano-4-hydroxycinnamic acid in 0.2% trifluoroacetic acid in acetonitrile-H_2_O (50%, v/v), and were externally calibrated using a mixture of peptide standards (Applied Biosystems).

N-terminal amino acid sequencing of *Eg*KU-1 was carried out by automatic Edman degradation on a pulsed liquid-phase sequencer (Applied Biosystems), at the laboratory of Dr Ulf Hellman (Ludwig Institute for Cancer Research, Uppsala Branch - Sweden).

Peptide mass fingerprinting of *Eg*KU-8 was performed by in-gel trypsin (Sequencing-grade, Promega) treatment of an SDS-PAGE band of the purified inhibitor followed by MALDI-TOF MS of the tryptic digest (4800 MALDI TOF-TOF Analyzer System, Applied Biosystems). *Eg*KU-8 was reduced and alkylated with iodoacetamide prior to treatment with the enzyme and peptides were extracted from the gel in 60% acetonitrile in 0.2% trifluoroacetic acid, concentrated by vacuum-drying and desalted using C18 reverse phase micro-columns (OMIX Pipette tips, Varian). Confirmation of the sequence of selected peptides was performed by collision-induced dissociation MS/MS experiments.

### Analysis of protoscolex secretions

Aliquots of about 100 µl of freshly isolated protoscoleces (roughly 50,000 larval worms of >95% viability, estimated by eosin exclusion) were incubated in 1 ml of RPMI 1640 containing penicillin/streptomycin (Sigma) for 3 h at 37°C with gentle agitation. Some aliquots were treated with 0.5 mg/ml of pepsin (Sigma) at pH 2 for 30 min at 37°C, washed and then incubated with RPMI for 3 h. Parasite viability was checked at the end of the cultures and found to have remained unchanged. The supernatants containing parasite secretions were kept at −70°C and analyzed by MALDI-TOF MS as described. The samples were concentrated and desalted by adsorption onto a reverse phase micro-column; and were eluted with matrix solution directly on the MALDI sample plate.

### Assays of protease inhibition

The inhibitory activity of purified *Eg*KU-1 and *Eg*KU-8 was tested against bovine and canine chymotrypsins (EC 3.4.21.1) and trypsins (EC 3.4.21.4). Bovine enzymes were obtained from Sigma whereas canine proteases were purified from the pancreas of a dog that had passed away due to an accidental cause, according to the procedure of Waritani *et al*.[64]. The following peptidases were thus assayed (MEROPS - http://merops.sanger.ac.uk - identifiers are indicated in brackets): from *Bos taurus*, chymotrypsin A (S01.001) and trypsin 1 (cationic, S01.151); from *Canis familiaris*, trypsins 1 (cationic, S01.151) and 2 (anionic, S01.120), and chymotrypsin B (S01.152).

Prior to inhibition studies, proteolytic activity in enzyme preparations was determined with fluorogenic substrates using initial steady-state rate conditions at 37°C and pH 8. Assays (200 µl) were performed in black 96-well microplates (Costar, Corning Life Sciences). Enzymes and substrates were dissolved in 50 mM Tris-HCl, pH 8.0 containing 0.01% Triton X-100 (v/v), and reactions were initiated by the addition of enzyme. The changes in fluorescence intensity, corresponding to the formation of the hydrolysis product 7-amino-4-methylcoumarin (AMC), were registered at excitation and emission wavelengths of 390 and 460 nm, respectively, with a microplate fluorescence reader (FLUOstar^*^ OPTIMA, BMG Labtechnologies). For trypsin activity, the artificial substrate N-*t*-BOC-Ile-Glu-Gly-Arg-AMC was used and for chymotrypsin, Suc-Ala-Ala-Pro-Phe-AMC. Calibration curves using AMC were carried out in each experiment. Initial steady-state rates of substrate hydrolysis were calculated from the linear portion of product (AMC) *versus* time plots when less than 10% of substrate had been consumed.

Protein concentrations of enzyme preparations and purified inhibitors were determined with the BCA reagent (Pierce) using bovine serum albumin as standard; and the active site concentration of trypsins and bovine chymotrypsin A by specific titration with the high affinity inhibitor BPTI [39], [65]. The active site concentration of canine chymotrypsin could not be estimated because, similar to bovine chymotrypsin B [23], it was not inhibited by BPTI.

The kinetic parameters for substrate and enzyme pairs were calculated from the non-linear fitting to the Michaelis-Menten equation. The values determined with the substrates specified above were, for canine proteases: *K_M_* = 25±3 µM and k_Cat_ = 38±2 s^−1^ for anionic trypsin; *K_M_* = 31±4 µM and k_Cat_ = 43±2 s^−1^ for cationic trypsin; and *K_M_* = 39 µM±2 s^−1^ for chymotrypsin B (k_Cat_ was not calculated because of the unknown active site concentration). And for the bovine enzymes: *K_M_* = 85±9 µM and k_Cat_ = 50±6 s^−1^ for cationic trypsin and *K_M_* = 30±2 µM and k_Cat_ = 19±2 s^−1^ for chymotrypsin A.

For inhibition studies, each of the enzymes was incubated with purified *Eg*KU-1 or *Eg*KU-8 for 15 min at 37°C prior to the addition of the appropriate fluorogenic substrate, to allow for the equilibration of the enzyme-inhibitor complexes. The substrate concentration (5 µM) was chosen so as to be well below the corresponding *K_M_*, as specified above.

### Inhibition studies with *Eg*KU-8

#### Tight-binding kinetics

In order to determine the inhibition constants (*K*_I_^*^) of *Eg*KU-8 towards canine trypsins (anionic and cationic) and bovine trypsin and chymotrypsin A, the initial steady-state rates of substrate hydrolysis in the presence of increasing concentrations of *Eg*KU-8 were measured after pre-incubation of the enzyme with inhibitor. The inhibition constants were calculated by nonlinear fitting to the Morrison equation for tight binding inhibitors [66], [67]:
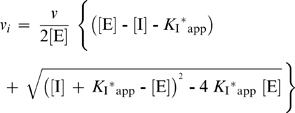
(1)where *K*_I_^*^_app_ is the apparent global dissociation constant of the enzyme-inhibitor complex, *v_i_* is the inhibited steady-state rate, *v* is the uninhibited rate, [I] is the total *Eg*KU-8 concentration and [E] is the total enzyme concentration. The true inhibition constants, *K*_I_^*^, were corrected from *K*_I_^*^_app_ according to the equation 2 for competitive inhibitors:
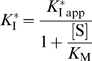
(2)


#### Slow-binding kinetics

The decrease in the rate of product formation during the first minutes after mixing the enzyme with *Eg*KU-8 and substrate (5 µM) was studied for increasing inhibitor concentrations. Progress curves were analyzed using the equation 3 [68] that describes the slow establishment of equilibrium between the enzyme and the inhibitor according to:
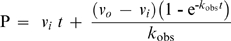
(3)where P is the concentration of AMC produced by hydrolysis of the substrate, *v_o_* is the initial rate, *v_i_* is the final steady-state rate and *k*_obs_ represents the apparent first order rate constant. Computer fitting of progress curves estimated values for *v_o_, v_i_* and *k*_obs_.

For Kunitz inhibitors that bind to the enzyme rapidly and reversibly forming an initial “loose” complex EI that isomerizes slowly to the final complex EI*, the reaction mechanism can be represented by the following scheme:
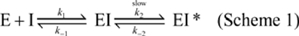



In this mechanism, the value of the apparent rate constant (*k*_obs_) is related to the kinetic constants *k*_2_ and *k*_−2_ and to the equilibrium dissociation constant of the initial loose complex *K*_I_ (*K*_I_ = *k_−1_/k_1_*), by equation 4 [69]:
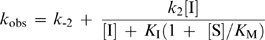
(4)


The constants *k_2_* and *K_I_* were determined from plots of *k*_obs_ vs [I], by computer fitting to equation 4. Because *k_-2_* was too small to be accurately estimated from these plots, it was determined using equation 5 [24] and data from situations where the ratio *v_i_/v_o_* was higher than 0.05:
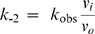
(5)


The values of *k_−2_*, *k_2_* and *K_I_* thus determined allowed to corroborate the inhibition constant K_I_^*^, according to equation 6:
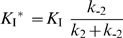
(6)


#### Data analysis

Computer fitting to non-linear equations was performed using the software Origin (OriginLab). All experiments were carried out at least two to three independent times and results shown are averages ± the standard error unless otherwise specified.

## Supporting Information

Table S1 The *E. granulosus* Kunitz protein family.
(0.04 MB PDF)

Figure S1 Phylogenetic analysis of *E. granulosus* and related Kunitz proteins from platyhelminths. The mature protein sequences predicted for *Eg*KU-1 - *Eg*KU-8, together with those identified among *E. multilocularis*, *T. solium* and planarian (*D. ryukuyensis* and *S. mediterranea*) ESTs were aligned with Clustal W2 [70]. A neighbor joining tree was constructed using MEGA4 [73] with default parameters. *E. multilocularis* (Em) and *T. solium* (Ts) sequences are as in Figure 1B; Sm-1 was deduced from DN300487 and DN307650 (derived from the same transcript); and Dr-1, from BW635664 in dbEST (refer to Table S1 for further details).
(0.89 MB TIF)

Figure S2 Confirmation of *Eg*KU-8 as the component of the minor rpHPLC peak. A pool of the fractions eluting around 24% ACN was resolved by SDS-PAGE and Coomassie-stained; the 7 kDa band was in-gel digested with trypsin, after reduction and alkylation with iodoacetamide. Tryptic fragments identified by MALDI-TOF MS provided 73% coverage (42/57 amino acids) of the sequence predicted for mature *Eg*KU-8. Peptides 1-15 and 16–20 were further verified by MS/MS experiments. The spectrum of the 16–20 peptide (m/z 653.33) is shown: signals from N-terminal (b ions) and C-terminal (y ions) fragments confirmed the sequence AYFPR. P, F, R and Y indicate signals from the immoniun ions of the corresponding amino acids.
(0.45 MB TIF)
