# Supplementary material for: A Family of Diverse Kunitz Inhibitors from Echinococcus granulosus Potentially Involved in Host-Parasite Cross-Talk
Source: PLoS One. 2009 Sep 17;4(9):e7009. doi: 10.1371/journal.pone.0007009 (PMC2740865; doi:10.1371/journal.pone.0007009)
Supplement: Table S1 — The E. granulosus Kunitz protein family. (0.04 MB PDF) [file pone.0007009.s001.pdf]

<u>Table S1.</u> The *E. granulosus* Kunitz protein family.

Name	cDNA sequence data[a]				Predicted protein parameters[b]				Similar Kunitz domain[e]	Close platyhelminth proteins[e]
	Cluster ID (reads)	cDNA source	Accession numbers	Consensus (nt)	SP/MP (aa)	MW (Da)[c]	pI	charge		
***Eg*KU-1**	EGC000603 (6)	PS/peps/H^+	CN650596, CN650753, CN650941, CN652374, CN652433, BI244287	359	20/58[d]	6599.5	9.2	+6	sp **P00985** IVB2_HEMHA - [23-75] $1e^{-10}$ Venom Basic Inhibitor 2 *H. haemacathus*	-[g]
***Eg*KU-2**	EGC000598 (5)	PS/peps/H^+	CN650229, CN650436, CN650784, CN652059, BI244281	626 (long 3'UTR)	20/101	12258.8	8.8	+4	tr **Q8NT7L9** BOMMO - [28-81] 0.0009 Serine Protease Inhibitor 1 *Bombyx mori*	**EL746785**[h] - *T. solium* larva 85% identity (98/115 aa)
***Eg*KU-3**	EGC001164 (1)	PS/peps/H^+	BQ173695	354	18/57	6405.4	5.1	-1	sp **P31713** ISH1_STOHE - [23-73] $6e^{-13}$ Kunitz Inhibitor 1 *Stoichactis helianthus*	**XvEMa04137 -** *E. multilocularis* larva 98% identity (74/75 aa)
***Eg*KU-4**	EGC004311 (1)	PS/peps/H^+	CN652174	535 (long 5'UTR)	20/64	7288.4	10.1	+14	sp **P00978** AMBP_BOVIN - [25-75] $7e^{-12}$ Alpha-1-microglobulin inter-alpha-trypsin inhibitor light chain *Bos taurus*	**XvEMa03312 -** *E. multilocularis* larva 92% identity (78/84 aa)<br><br>**EL763407 -** *T. solium* adult 63% identity (53/84 aa)[i]
***Eg*KU-5**	EGC002977 (2)	PS<br><br>PS/peps/H^+	CN649551<br><br>CN650837	317	29/60	7239.3	9.6	+9	tr **O54819** TFPI_MOUSE - [37-87] $2e^{-12}$ Tissue factor pathway inhibitor 1 *Mus musculus*	**BW635664 -** *D. ryukyuensis* adult 60% identity (31/51 aa, KU domain)<br><br>**DN300487**[j] and **DN307650**[j] – *S. mediterranea* mature hermaphrodites 56% identity (29/51 aa, KU domain)
***Eg*KU-6**	EGC003000 (2)	PS	CN649590, CN649996	416	18/82	9778.9	6.9	0	tr **O45881** CAEEL - [24-74] $7e^{-12}$ Protein W01F3.3, partially confirmed *Caenorhabditis elegans*	**EL743839** - *T. solium* larva 75% ident. (75/100 aa)
***Eg*KU-7**	EGC002672 (1)	PS/peps/H^+	CN648947	327 (no 3'UTR)	20/79	9108.0	5.1	-3	sp **Q868Z9** PPN_ DROME- [25-75] $7e^{-12}$ Papilin (precursor) *D. melanogaster*	-[g]
***Eg*KU-8**	-	PS/peps/H^+	FJ031017	228	18/57[d]	6519.4	7.8	+1	tr **Q8WPI2** BOOMI - [23-73] $1e^{-12}$ *Boophilus microplus* boophilin	**XvEMa16368 -** *E. multilocularis* larva 98% identity (74/75 aa)

[a]For *Eg*KU-1-*Eg*KU-7, identified while surveying the protoscolex transcriptome, data include: i) the EST cluster identification in PartiGeneDB (http://www.compsysbio.org/partigene/), EGC00XXXX and the number of reads in the cluster; ii) the libraries from which ESTs were derived, designed as: PS, untreated protoscoleces; PS/peps/H^+, protoscoleces treated with pepsin at pH 2; iii) the NCBI accession number of ESTs in the cluster; iv) the size and relevant features of the consensus sequence (except for *Eg*KU-7, this accounts for the full-length cDNA). For *Eg*KU-8, data include: i) the source of RNA used for RT-PCR; ii) the NCBI accession number of the cDNA cloned on the basis of the putative *E. multilocularis* orthologue, as detailed in the text; iii) the size of the full coding sequence thus isolated.

[b]Analyses were carried out with the tools available at the ExPASy Proteomics Server (http://expasy.org.ch/): “Translate”, for prediction of protein sequences; “SignalP”, for prediction of signal peptides (SP), signal peptidase cleavage sites and, correspondingly, mature proteins (MP); and “ProtParam”, for calculation of the theoretical MW (average) and pI of predicted mature proteins; the charge was estimated as the difference between positively and negatively charges amino acids. The consensus sequence of clustered ESTs was used for *Eg*KU-1-*Eg*KU-7.

[c]The theoretical MW of predicted mature proteins (estimated as detailed in b) minus 6 Da is indicated, under the assumption that the six conserved Cys residues of the Kunitz domains form disulphide bridges in native proteins.

[d]The experimentally determined N-terminus of the native protein directly isolated from protoscoleces confirmed the SignalP prediction.

[e]The predicted full-length *Eg*KU-1-*Eg*KU-8 were analyzed by InterProScan at ExPASy and found to hit InterPro 002223: Proteinase inhibitor I2, Kunitz metazoa. The Kunitz domain showing the highest score in PD000222 was recorded for every protein. Data include: the accession number in Swiss-Prot (sp) or TrEMBL (tr) and name of the protein bearing the domain, the score and the similarity region in the *E. granulosus* protein.

[f]Related platyhelminth proteins were identified blasting the predicted full-length *Eg*KU-1-*Eg*KU-8 against ‘non-human, non-rodent ESTs’ in dbEST (option ‘est_others’ at the NCBI server), and *E. multilocularis* ESTs available at ‘Full-Echinococcus’ (http://fullmal.hgc.jp/em/docs/blast.html/). For each putative protein, data include: i) an identifier of the corresponding EST - the NCBI accession number for *T. solium*, *D. ryukyuvensis* and *S. mediterranea*; and the clone name in Full-Echinococcus for *E. multilocularis*; ii) the cDNA library from which each EST was derived; iii) the level of identity with the corresponding *E. granulosus* protein and the length of the alignment. In the case of *Eg*KU-5, identity is over the 51 aa of the Kunitz domain (between conserved Cys). Only the predicted protein showing the highest similarity with every *E. granulosus* molecule is indicated for each platyhelminth.

[g]Proteins close to *Eg*KU-1and *Eg*KU-7 were the same as for *Eg*KU-4 and *Eg*KU-6, respectively, but the identity level was lower than for the latter.

[h]EST does not contain the full coding sequence: as shown in Fig 1b (*Ts*-2), the sequence translated from EL746785 has no starting Met.

[i]Two additional *T. solium* ESTs with about 60% identity with EgKU-4 were identified: EL763445 (adult) and EL740737 (larva).

[j]These ESTs were assigned to transcript mk4.029059.00.01 in the *S. mediterranea* Genome Database (SmedGD: http://smedgd.neuro.utah.edu).
