# Supplementary figures and images for: A Family of Diverse Kunitz Inhibitors from Echinococcus granulosus Potentially Involved in Host-Parasite Cross-Talk
Source: PLoS One. 2009 Sep 17;4(9):e7009. doi: 10.1371/journal.pone.0007009 (PMC2740865; doi:10.1371/journal.pone.0007009)

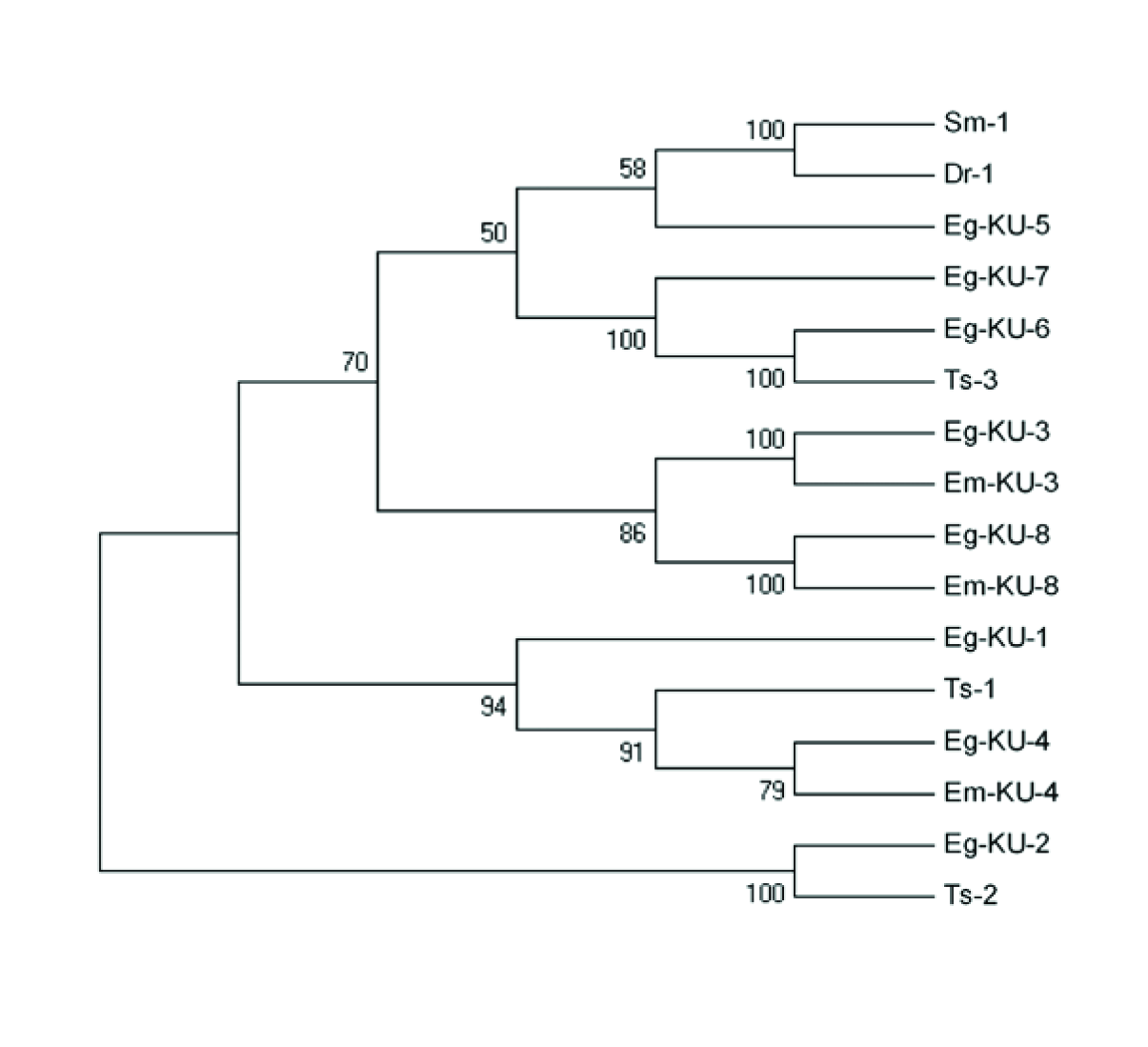

Supplement: Figure S1 — Phylogenetic analysis of E. granulosus and related Kunitz proteins from platyhelminths. The mature protein sequences predicted for EgKU-1 - EgKU-8, together with those identified among E. multilocularis, T. solium and planarian (D. ryukuyensis and S. mediterranea) ESTs were aligned with Clustal W2 [70]. A neighbor joining tree was constructed using MEGA4 [73] with default parameters. E. multilocularis (Em) and T. solium (Ts) sequences are as in Figure 1B; Sm-1 was deduced from DN300487 and DN307650 (derived from the same transcript); and Dr-1, from BW635664 in dbEST (refer to Table S1 for further details). (0.89 MB TIF) [file pone.0007009.s002.tif]

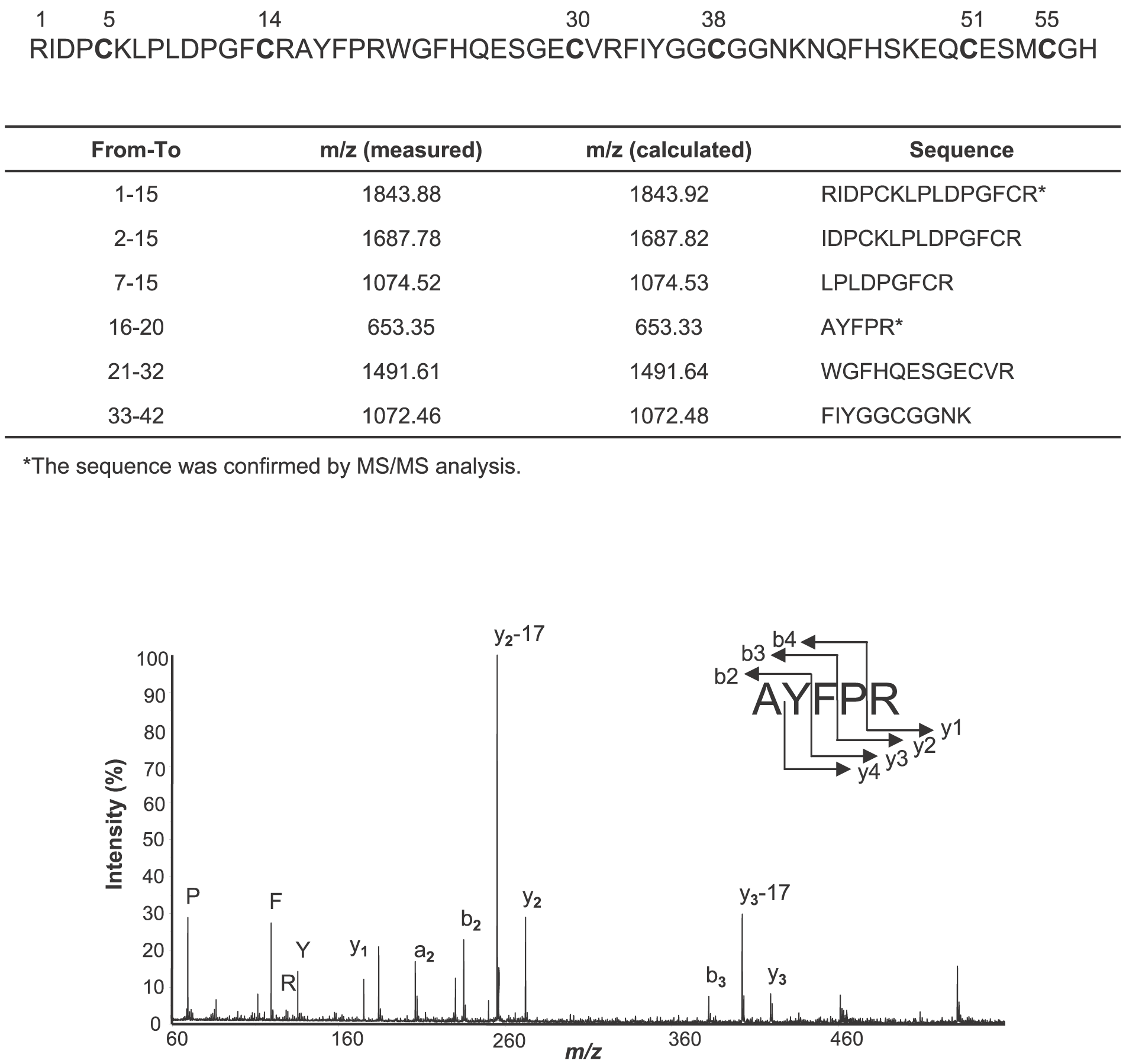

Supplement: Figure S2 — Confirmation of EgKU-8 as the component of the minor rpHPLC peak. A pool of the fractions eluting around 24% ACN was resolved by SDS-PAGE and Coomassie-stained; the 7 kDa band was in-gel digested with trypsin, after reduction and alkylation with iodoacetamide. Tryptic fragments identified by MALDI-TOF MS provided 73% coverage (42/57 amino acids) of the sequence predicted for mature EgKU-8. Peptides 1-15 and 16–20 were further verified by MS/MS experiments. The spectrum of the 16–20 peptide (m/z 653.33) is shown: signals from N-terminal (b ions) and C-terminal (y ions) fragments confirmed the sequence AYFPR. P, F, R and Y indicate signals from the immoniun ions of the corresponding amino acids. (0.45 MB TIF) [file pone.0007009.s003.tif]
